# The effect of a pre- and post-operative exercise program versus standard care on physical activity and sedentary behavior of patients with esophageal and gastric cancer undergoing neoadjuvant treatment prior to surgery (the PERIOP-OG Trial): a randomized controlled trial^†^

**DOI:** 10.1093/dote/doae044

**Published:** 2024-05-20

**Authors:** Lisa Loughney, Kate Murphy, Roisin Tully, William B Robb, Noel McCaffrey, Kieran Dowd, Fiona Skelly

**Affiliations:** ExWell Medical, Dublin, Ireland; School of Health & Human Performance, Dublin City University, Dublin, Ireland; The Royal College of Surgeons in Ireland, Dublin, Ireland; School of Health & Human Performance, Dublin City University, Dublin, Ireland; The Royal College of Surgeons in Ireland, Dublin, Ireland; Department of Upper GI Surgery, Beaumont Hospital, Dublin, Ireland; The Royal College of Surgeons in Ireland, Dublin, Ireland; Department of Upper GI Surgery, Beaumont Hospital, Dublin, Ireland; ExWell Medical, Dublin, Ireland; SHE Research Group, Department of Sports & Health Science, Technological University of the Shannon, Athlone Westmeath, Ireland; ExWell Medical, Dublin, Ireland; SHE Research Group, Department of Sports & Health Science, Technological University of the Shannon, Athlone Westmeath, Ireland

**Keywords:** exercise training, neoadjuvant chemoradiotherapy, physical activity, prehabilitation, sedentary behavior

## Abstract

Neoadjuvant cancer treatment (NCT) reduces both physical fitness and physical activity (PA) levels, which can increase the risk of adverse outcomes in cancer patients. This study aims to determine the effect of exercise prehabilitation on PA and sedentary behavior (SB) in patients undergoing NCT and surgery for esophagogastric malignancies. This study is a randomized pragmatic controlled multi-center trial conducted across three Irish hospitals. Participants were aged ≥18 years scheduled for esophagectomy or gastrectomy and were planned for NCT and surgery. Participants were randomized to an exercise prehabilitation group (EX) that commenced following cancer diagnosis, continued to the point of surgery, and resumed following recovery from surgery for 6 weeks or to usual care (UC) who received routine treatment. The primary outcome measures were PA and SB. Between March 2019 and December 2020, 71 participants were recruited: EX (*n* = 36) or UC (*n* = 35). No significant differences were found between the EX group and UC group on levels of PA or SBs across all measured timepoints. Significant decreases in moderate–vigorous physical activity levels (MVPAs) were found between baseline and post-surgery (*P* = 0.028), pre-surgery and post-surgery (*P* = 0.001) and pre-surgery and 6-week follow-up (*P* = 0.022) for all participants. Step count also significantly decreased between pre-surgery and post-surgery (*P* < 0.001). Baseline aerobic fitness was positively associated to PA levels and negatively associated with SB. Esophagogastric cancer patients have lower than recommended levels of PA at the time of diagnosis and this decreased further following completion of NCT. An optional home- or group-based exercise intervention was not effective in improving PA levels or behaviors across the cancer treatment journey.

## INTRODUCTION

Multimodal treatment, including neoadjuvant cancer treatment (NCT) and surgery, is commonly used as an adjunctive therapy in the treatment of oesophageal and gastric cancer.^1^^,^^2^ Despite an increase in survival rates following multimodal treatments,^1^^,^^2^ perioperative (at or around time of surgery) and post-operative morbidity is still high.^3^^,^^4^ NCT reduces both physical fitness and physical activity (PA), which is linked to increased risk of perioperative morbidity.^5^^,^^6^ Poor physical fitness reflects a reduced physiological reserve and is linked with post-operative complications.^7^ However, little is known about the impact of different phases of treatment on PA levels in this cohort.

High levels of physical fitness and PA pre-surgery are associated with better post-operative outcomes and lower risk of cancer-specific and overall mortality.^8^ In 2019, the American College of Sports Medicine published recommendations that PA can reduce the risk of breast, colon, and prostate cancer. Increasing PA following a cancer diagnosis reduces cancer-specific death in those with breast cancer and death from any cause in colorectal cancer.^9^ The current recommendations for PA in people with cancer include a minimum of 150 minutes per week of moderate to vigorous PA and strengthening exercises. The pre-operative setting provides the opportunity to initiate an exercise program that may possibly influence PA^10^ and sedentary behavior (SB). Although there is a growing evidence base on the beneficial role of prehabilitation, few studies report PA behavior outcomes assessed via device-based measures. One study previously reported reduced daily step count, energy expenditure and metabolic equivalent (MET) following NCT in a cohort of colorectal cancer patients.^11^ However, to our knowledge, no study has reported PA or SB levels in patients undergoing the complex treatment pathway of NCT and surgery for esophagogastric malignancies.

This study aimed to determine the effect of a community-based exercise training program compared to usual care (UC) (without formal exercise intervention) of patients undergoing NCT and surgery for esophagogastric cancers on daily PA and SB.

## METHODS

### Study design

This was a nested study of a larger randomized, pragmatic, controlled, multi-center, trial (PERIOP-OG Trial). Participants were recruited at three university teaching hospitals. The protocol is published elsewhere.^12^ The methodology for the PERIOP-OG trial was based on experience gained from a previous feasibility study and was informed by patient and public representatives. The study was registered at ClinicalTrials.gov with trial registration number NCT03807518; the date of first registry was January 17, 2019.

### Participants and randomization

Inclusion criteria for this study were: patients aged ≥18 years planned for neoadjuvant chemotherapy or neoadjuvant chemoradiotherapy prior to esophagectomy or gastrectomy at any of the recruiting hospitals. Eligible patients were identified at multi-disciplinary cancer meetings, given oral information along with an information leaflet, and were then contacted 72 hours later to confirm participation. A baseline assessment visit was scheduled where informed consent was obtained. Participants were randomized using central random allocation sequence (1:1).

### Procedures

All patients were asked to wear a PA monitor (activPAL^3^ micro [PAL Technologies Ltd. Glasgow, Scotland]) at five different time points for 7 days: baseline/pre-NCT, post-NCT, pre-surgery, post-surgery, and 6 weeks later.

#### Usual care

The usual care (UC) group received routine care throughout their cancer pathway. No specific advice about exercise training was offered.

#### Exercise prehabilitation

The exercise-training program started before NCT (if time allowed), continued throughout NCT, and following completion of NCT up to the point of surgery and resumed for 6 weeks after surgery once patients were deemed clinically fit. Participants in exercise prehabilitation (EX) were offered an option to participate in either a center-based exercise program (CBEP) (in any of the seven exercise centers across Ireland lead by ExWell Medical) or a home-based exercise program (HBEP). All participants in EX were provided with an exercise program pack, which included a manual exercise handbook, a Fitbit, a rate of perceived exertion scale, and a PA diary. They were also provided with a link to an online motivational video developed specifically for the PERIOP-OG trial. The exercise training program is reported elsewhere.^12^

### Outcomes

#### Physical activity behaviors

The activPAL^3^ micro was used to quantify free-living activity behaviors. The device measures bodily accelerations using a triaxial accelerometer sampling at 20 Hz for 15-second (15-s) epochs. Proprietary algorithms detect thigh accelerations to accurately determine postural orientation of the wearer.^13^ The device was worn on the midpoint of the anterior aspect of the right thigh. It was covered with a water-resistant nitrile sleeve and attached to the skin using a 3M Tegaderm Film (Kooperationspartner Wundversorgung, Germany) adhesive dressing. Participants were instructed to wear the device continuously, 24 hours per day, for 7 consecutive days, except during water immersion activities (i.e. swimming and bathing). The activPAL monitors were issued at each time point either at the in-person assessment or via post. Standardized detailed wear protocols were provided either in-person and or via post.

#### Accelerometer data processing

Raw acceleration data were processed and stored as 15-s epoch summary files. Proprietary algorithms classified activities into sitting/lying time, standing time, stepping time, step count, and activity counts. Tudor-Locke *et al*. defined 100 steps/minute as the threshold for MVPA in older adults.^14^ Based off this threshold, MVPA was classified as ≥25 steps within a 15-s epoch. A valid day was defined as ≥600 minutes of recording during daytime hours.^15^ Non-wear time was defined as ≥60 minutes of consecutive zero accelerometer counts.^13^

SB characteristics were examined using a customized MATLAB® (version 7.0.1, The Mathworks Inc, Natick, MA, USA) software program.^13^ The MATLAB program examined the sedentary output file of the activPAL^3^ micro accelerometer epoch-by-epoch. The program identified sedentary periods as follows; a change in inclination from upright to sitting was identified as the start of the sedentary period; the sedentary period ended when a change in inclination from sitting to upright occurred. This approach enabled the quantification of all sitting bouts. The accumulated time spent in sitting bouts identified total sedentary time. The program also examined the sequence of each sedentary period to identify the start and the end time for each one in hh:mm:ss format. Sleep time for each participant was manually identified by examination of the sedentary epoch Microsoft Excel output produced by MATLAB. The first break in a SB after 6 am each day was selected as the rise time. The last registered non-sedentary epoch of the day, which was followed by a prolonged, uninterrupted sedentary period (>2 hours) was selected as the time the subject went to bed. This method allowed for the differentiation between sleep time and daily waking SB. Sedentary bouts were categorized by specific durations, namely, <10 minutes, 11–30 minutes, 31–60 minutes, and >60 minutes The number and duration of daytime sedentary bouts for each category were calculated.

### Statistical analysis

Statistical analyses were performed using IBM SPSS Statistics 28 (IBM, Armonk, NY, USA). An independent *t*-test was used to compare differences between the intervention and control group at baseline. Difference-in-difference (DID) assessments were conducted for group difference changes in outcomes between baseline/pre-NCT and pre-surgery and between post-surgery and 6 weeks later using an independent *t*-test. To investigate longitudinal changes in all repeated measure variables, linear mixed-model analysis (MMA) was used. The model was analyzed for autoregressive, compound symmetry, diagonal, toeplitz, and unstructured variance structures. The Akaike information criterion (AIC) and the Bayesian information criterion (BIC) were used as a metric to identify the best-fit model. Time was treated as repeated measures and incorporated as a fixed effect in the model. The main effects for time and time*group interaction were investigated. To control for heterogeneity within the population, models were adjusted for baseline 6-minute timed trial (6MTT) results. To determine the timepoints at which intervention effects occurred, Bonferroni post-hoc stratified analysis comparing estimated marginal means at each timepoint were performed for outcomes that indicated a significant main effect for time.

## RESULTS

Between March 1, 2019 and December 31, 2020, 71 participants agreed to participate: 50 were recruited from Beaumont Hospital, 11 from Mercy University Hospital Cork and 10 from University Hospital Galway. Thirty-six were randomized to EX (following recruitment, one participant’s pathway changed and was therefore no longer eligible) and 35 to UC. The mean age was 62.8 years (standard deviation (SD) = −9.2), and 73% were male. Patient characteristics are presented in Table 1.

**Table 1:** Baseline characteristics

**Patient characteristics**	**Exercise** **(***n* = **36)**	**Usual care** **(***n* = **35)**	**All patients** **(***n* = **71)**	**P value**
**Age** (years)^§^	62.8 (9.2)	61.5 (8.8)	62.2 (9.0)	0.53
**Gender** ^¥^				
Male	27 (75)	25 (71)	52 (73)	
Female	9 (25)	10 (29)	19 (27)	0.73
**Body mass index** (kg/m^2^)^§^	27.9 (5.5)	27.7 (4.5)	27.8 (5)	0.91
**Frailty score** ^§^	25.7 (4.1)	27.2 (5.3)	26.4 (4.8)	0.19
**Smoking status** ^¥^				
Current	3 (9)	5 (14)	8 (11)	
Previous	16 (44)	17 (49)	33 (47)	
Never	16 (44)	13 (37)	29 (41)	
Unknown	1 (3)	0 (0)	1 (1)	0.66
**Dysphagia score** ^¥^				
0	12 (33)	17 (49)	29 (41)	
1	8 (22)	7 (20)	15 (21)	
2	7 (19)	4 (11)	11 (16)	
3	6 (17)	6 (17)	12 (17)	
4	1 (3)	0 (0)	1 (1)	
Unknown	2 (6)	1 (3)	3 (4)	0.6
**Nutrition** ^¥^				
I	20 (56)	16 (46)	36 (51)	
II	6 (17)	7 (20)	13 (18)	
III	8 (22)	11 (31)	19 (27)	
Unknown	2 (5)	1 (3)	3 (4)	0.61
**ECOG score** ^¥^				
0–1	34 (94)	32 (91)	66 (93)	
2–3	2 (6)	3 (9)	5 (7)	0.25
**ASA grade** ^¥^				
I	6 (17)	1 (3)	7 (10)	
II	18 (50)	25 (71)	43 (60)	
III	12 (33)	9 (26)	21 (30)	0.08
**Tumor location** ^¥^				
Esophageal	26 (72)	18 (51)	44 (62)	
Junctional	6 (17)	7 (20)	13 (18)	
Gastric	4 (11)	10 (29)	14 (20)	0.18
**cT category** ^**¥**^				
T1	1 (3)	1 (3)	2 (3)	
T2	6 (16)	2 (6)	8 (11)	
T3	26 (72)	26 (74)	52 (73)	
T4	1 (3)	4 (11)	5 (7)	
Unknown	2 (6)	2 (6)	4 (6)	0.42
**cN category** ^**¥**^				
N0	14 (39)	9 (25)	23 (32)	
N1	14 (39)	16 (46)	30 (42)	
N2	6 (17)	8 (23)	14 (20)	
Unknown	2 (5)	2 (6)	4 (6)	0.47
**Neoadjuvant treatment** ^¥^				
CROSS	29 (81)	23 (66)	52 (73)	
FLOT	6 (16)	12 (34)	18 (25)	
No treatment (change of pathway)	1 (3)	—	1 (2)	0.12
**Surgical characteristics** ^¥^	**Exercise (** *n* = **29)**	**Usual care (** *n* = **25)**	**All patients (** *n* = **54)**	
Surgery type				
* Esophagectomy*	25 (86)	15 (60)	40 (74)	
* Gastrectomy*	4 (14)	10 (40)	14 (26)	0.01^*^
Surgical procedure				
Esophagectomy				
*Transhiatal*	*2 (8)*	*0 (0)*	2 (4)	
*Ivor Lewis*	*22 (88)*	*15 (100)*	37 (93)	
*McKeown*	*1 (4)*	*0 (0)*	1 (3)	0.47
*No. of minimally invasive approaches*	19 (76)	9 (60)	28 (70)	0.16
Gastrectomy				
*Total gastrectomy*	2 (50)	3 (30)	5 (36)	
*Extended total*	*0 (0)*	*2 (20)*	2 (14)	
*Partial gastrectomy*	*2 (50)*	*5 (50)*	7 (50)	0.41
**Location of anastomosis** ^¥^				
Cervical	3 (12)	0 (0)	3 (7)	
Thoracic	22 (88)	15 (100)	37 (93)	0.04^*^
**Thoracic phase** ^¥^				
Open	4 (16)	6 (40)	10 (25)	
Thoracoscopic completed	19 (76)	9 (60)	28 (70)	
Not applicable (transhiatal)	2 (8)	0	2 (5)	0.16
**Abdominal phase** ^¥^				
* Open*	3 (12)	3 (20)	6 (15)	
* Lap converted to open*	1 (4)	1 (7)	3 (7)	
* Lap completed*	12 (48)	6 (40)	18 (45)	
* Lap assisted*	3 (12)	0 (0)	2 (5)	
* Robotic*	6 (24)	5 (33)	11 (28)	0.53
**Gastrectomy**: **surgical access**	**Exercise (** *n* = **4)**	**Usual care (** *n* = **10)**	**All patients (** *n* = **12)**	
*Lap completed*	4 (100)	8 (80)	12 (86)	
*Lap converted to open*	0 (0)	2 (20)	2 (14)	
**LOT-R** ^§^	21.8 (3)	18.1 (7)	20 (5.6)	0.00^*^

Data are presented as mean (SD)^§^ and *n* (%)^¥^. ^*^ was taken as statistically significant (*P* < 0.05). Abbreviations: ASA, American Society of Anaesthesiology; ECOG, Eastern Cooperation Oncology Group; LOT-R, Revised Life Orientation Test Questionnaire.

Note: Dysphasia is classified as follows: 0—able to eat normal diet/no dysphagia, 1—able to swallow some solid foods, 2—able to swallow only semi solid foods, 3—able to swallow liquids only, 4—unable to swallow anything/total dysphagia. Nutrition is classified as follows: 1—no additional supplementation needed, 2—PO supplements and referred to dietician, 3—early dietetic intervention with assessment for supplement food feeding. ASA is classified as follows: 1—a normal, healthy patient, 2—a patient with mild systemic disease, 3—a patient with severe, systemic disease, 4—a patient with severe, systemic disease that is a constant threat to life, 5—a moribund patient who is not expected to survive without the operation, 6—a declared brain-dead patient whose organs are being removed for donation. ECOG is classified as follows: 0—fully active, able to carry on all pre-disease performance without restriction, 1—restricted in physically strenuous activity but ambulatory and able to carry out work of a light or sedentary nature, e.g. light house work, office work, 2—ambulatory and capable of all selfcare but unable to carry out any work activities; up and about more than 50% of waking hours, 3—capable of only limited selfcare; confined to bed or chair more than 50% of waking hours, 4—completely disabled; cannot carry out any selfcare; totally confined to bed or chair, 5—dead.

### Activity behavior

Measures of PA and SB for the EX group and the UC groups are displayed in Table 2. There were no significant differences between the EX groups and the UC groups on measures of PA or SB at baseline. No significant DID were found between baseline/pre-NCT and pre-surgery or between post-surgery and 6 weeks later between the EX group and UC group. Table 3 presents results of MMA. Statistically significant main effects for time were found for standing hours (*P* < 0.001, Cohen’s *D* = 0.33), MVPA (*P* < 0.001, Cohen’s *D* = 0.26) and daily step count (*P* = 0.017, Cohen’s *D* = 0.44). There was a significant difference in standing hours between post NCT and pre-surgery (Δ = 1.6 ± 0.45 hours, *P* = 0.011) and between pre-surgery and post-surgery (Δ = −1.5 ± 0.37 hours, *P* = 0.003). There was a significant difference in MVPA hours between baseline and post-surgery (Δ = -0.16 ± 0.05, *P* = 0.028), pre-surgery to post-surgery (Δ = −0.18 ± 0.05, *P* = 0.001) and pre-surgery to 6 week follow-up (Δ = −0.17 ± 0.05, *P* = 0.022). Step count significantly decreased between pre-surgery and post-surgery (Δ = −1493 ± 504, *P* < 0.001).

**Table 2:** Activity behavior of EX and UC groups

		**Baseline, *n***	**Post-NCT, *n***	**Pre-surgery, *n***	**Post-surgery, *n***	**6-Week reassessment, *n***	**Diff-In-diff** **baseline to pre-surgery,** ***P*-value**	**Diff-In-diff** **post-surgery to 6 weeks,** ***P*-value**
**Physical activity**								
Standing (hours)								
	Exercise	3.4 (1.4), 29	2.9 (1.2), 25	4.2 (2.1), 18	2.8 (1.0), 21	3.0 (1.3), 21	−1.07, 0.174	−0.02, 0.962
	Control	3.2 (1.7), 27	3.4 (1.4), 19	4.6 (3.2), 19	3.1 (1.5), 18	3.7 (1.7), 17		
Stepping (hours)								
	Exercise	1.5 (0.8), 29	1.4 (0.6), 25	1.5 (0.9), 18	1.2 (0.6), 21	1.2 (0.5), 21	−0.15, 0.565	−0.15, 0.448
	Control	1.4 (0.8), 27	1.3 (0.7), 19	1.5 (0.6), 19	1.5 (0.9), 18	1.6 (1.0), 17		
LIPA (hours)								
	Exercise	1.0 (0.5), 29	0.9 (0.4), 25	1.1 (0.5), 18	0.9 (0.4), 21	1.0 (0.4), 21	0.06, 0.726	−0.11, 0.389
	Control	1.1 (0.6), 27	1.1 (0.5), 19	1.1 (0.5), 19	1.3 (0.8), 18	1.4 (0.9), 17		
MVPA (hours)								
	Exercise	0.4 (0.4), 29	0.4 (0.4), 25	0.4 (0.4), 18	0.3 (0.3), 21	0.3 (0.3), 21	−0.06, 0.558	−0.08, 0.440
	Control	0.3 (0.3), 27	0.2 (0.2), 19	0.4 (0.3), 19	0.2 (0.2), 18	0.2 (0.2), 17		
Daily step count								
	Exercise	7724.1 (4151.3), 29	6692.5 (3363.7), 25	7326.8 (4882.3), 19	5873.6 (3676.1), 21	5954.6 (3027.0), 21	−112.0, 0.921	−1088.3, 0.316
	Control	6589.9 (4151.3), 27	5802.9 (3299.5), 19	7206.3 (2831.6), 19	6831.3 (3725. 5), 18	6871.7 (4120.9), 17		
**Sedentary behavior**								
*No. sedentary bouts <10 minutes*								
	Exercise	29.1 (10.9), 29	29.8 (11.8), 25	32.2 (15.9), 17	31.4 (16.0), 20	29.2 (13.5), 21	4.95, 0.221	−2.14, 0.476
	Control	28.3 (11.9), 26	31.7 (12.6), 19	29.9 (12.7), 19	29.3 (12.0), 18	29.2 (11.0), 17		
*Time in sedentary bouts <10 minutes*								
	Exercise	84.0 (30.5), 29	91.2 (36.1), 25	98.1 (46.0), 17	93.6 (44.3), 20	84.7 (37.7), 21	15.72, 0.060	−8.97, 0.313
	Control	83.8 (36.6), 26	92.9 (35.9), 19	84.1 (29.4), 19	84.8 (30.8), 18	86.2 (33.6), 17		
*No. of sedentary bouts <20 minutes*								
	Exercise	35.3 (11.9), 29	36.4 (13.4), 25	38.4 (17.8), 17	37.7 (16.6), 20	35.4 (14.0), 21	4.83, 0.280	−2.49, 0.453
	Control	34.5 (12.7), 26	38.3 (14.0), 19	36.0 (14.0), 19	35.6 (12.7), 18	35.5 (12.1), 17		
*Time in sedentary bouts < 20 minutes*								
	Exercise	174.4 (54.5), 29	184.6 (67.5), 25	187.4 (79.4), 17	183.3 (57.9), 20	172.4 (48.8), 21	11.67, 0.512	−15.94, 0.298
	Control	173.1 (52.4), 26	187.6 (65.8), 19	173.1 (53.6), 19	174.5 (50.1), 18	175.8 (58.8), 17		
*No. sedentary bouts 11–30 minutes*								
	Exercise	9.5 (2.8), 29	9.7 (3.8), 25	9.5 (4.5), 17	9.2 (2.3), 20	9.5 (2.0), 21	−0.38, 0.719	0.14, 0.867
	Control	9.1 (2.2), 26	10.0 (3.5), 19	9.1 (2.7), 19	9.5 (2.7), 18	9.5 (3.4), 17		
*Time in sedentary bouts 11–30 minutes*								
	Exercise	168.4 (47.8), 29	170.2 (66.5), 25	168.5 (80.8), 17	161.2 (37.5), 20	169.6 (33.9), 21	−12.28, 0.505	5.12, 0.737
	Control	158.4 (38.5), 26	178.6 (60.7), 19	161.0 (46.7), 19	167.9 (48.2), 18	167.5 (60.0), 17		
*No. of sedentary bouts 31–60 minutes*								
	Exercise	4.1 (1.1), 29	3.8 (1.2), 25	3.9 (1.4), 17	3.8 (1.2), 20	3.9 (1.0), 21	−0.49, 0.417	−0.23, 0.512
	Control	3.8 (1.1), 26	3.5 (1.2), 19	3.9 (1.1), 19	4.1 (1.0), 18	4.0 (1.2), 17		
*Time in sedentary bouts 31–60 minutes*								
	Exercise	170.9 (45.2), 29	160.5 (45.7), 25	163.3 (55.9), 17	158.7 (50.8), 20	163.5 (44.0), 21	−26.37, 0.295	−6.44, 0.675
	Control	160.9 (46.5), 26	145.5 (45.9), 19	168.0 (46.0), 19	168.6 (41.2), 18	165.7 (50.3), 17		
*No. of sedentary bouts >60 minutes*								
	Exercise	2.3 (0.8), 29	2.5 (1.0), 25	2.1 (0.9), 17	20 (1.1), 20	2.7 (0.9), 21	−0.29, 0.509	0.23, 0.499
	Control	2.7 (1.3), 26	2.9 (1.4), 19	2.6 (1.2), 19	2.7 (1.1), 18	2.4 (1.2), 17		
*Time in sedentary bouts > 60 minutes*								
	Exercise	293.7 (206.9), 29	301.7 (270.7), 25	340.9 (401.4), 17	328.6 (159.3), 20	326.4 (125.5), 21	58.40, 0.657	−49.76, 0.529
	Control	411.6 (339.7), 26	400.4 (353.4), 19	312.4 (222.0), 19	316.9 (235.0), 18	356.4 (376.8), 17		
*No. of sedentary bouts > 90 minutes*								
	Exercise	1.0 (0.5), 29	1.3 (0.7), 25	1.3 (0.8), 17	1.3 (0.6), 20	1.3 (0.5), 21	−0.03, 0.904	0.13, 0.599
	Control	1.4 (0.9), 26	1.4 (0.9), 19	1.2 (0.7), 19	1.2 (0.9), 18	1.1 (0.8), 17		
*Time in sedentary bouts >90 minutes*								
	Exercise	198.3 (206.2), 29	217.6 (267.0), 25	276.1 (409.5), 17	223.9 (142.5), 20	221.5 (118.6), 21	77.11, 0.579	−62.72, 0.416
	Control	315.4 (330.8), 26	291.5 (356.0), 19	213.2 (209.3), 19	203.2 (232.9), 18	265.4 (375.4), 17		
*Total no. of sedentary bouts*								
	Exercise	44.9 (11.8), 29	45.9 (13.7), 25	47.7 (18.2), 17	47.2 (16.1), 20	45.3 (13.4), 21	3.79, 0.364	−2.00, 0.514
	Control	43.9 (12.2), 26	48.1 (13.3), 19	45.5 (13.4), 19	45.6 (12.5), 18	45.0 (12.0), 17		
*Total sedentary waking time (minutes)*								
	Exercise	717.0 (207.8), 29	723.6 (235.0), 25	770.8 (414.5), 17	742.1 (158.1), 20	744.2 (127.1), 21	0.59, 0.803	−1.00, 0.415
	Control	814.8 (340.3), 26	817.4 (306.3), 19	725.5 (203.1), 19	738.2 (193.6), 18	775.7 (342.3), 17		

Data are presented as mean (SD)^§^ and *n* (%)^¥^. Abbreviations: LIPA, light-intensity physical activity; MVPA, moderate- to vigorous-intensity physical activity.

**Table 3:** Activity behavior MMA

**Outcome measure**						**Time**		Time*Group		**BL 6MTT**	
	**BL**	**Post-NCT**	**Pre- surgery**	**Post-surgery**	**6 weeks**	***F***	**sig**	***F***	**sig**	***F***	**sig**
***Standing hours***	3.5 ± 0.2	3.0 ± 0.2	4.6 ± 0.4	3.1 ± 0.2	3.4 ± 0.2	7.5	**<.001**	0.1	.989	23.4	**<.001**
***Stepping hours***	1.5 ± 0.1	1.3 ± 0.1	1.5 ± 0.1	1.3 ± 0.1	1.4 ± 0.1	2.1	.079	0.5	.728	10.6	**.002**
***LIPA hours***	1.1 ± 0.1	1.1 ± 0.1	1.1 ± 0.1	1.1 ± 0.1	1.1 ± 0.1	1.0	.444	0.4	.824	7.4	**.009**
***MVPA hours***	0.4 ± 0.04	0.3 ± 0.04	0.4 ± 0.04	0.2 ± 0.04	0.2 ± 0.04	5.0	**<.001**	0.8	.557	19.9	**<.001**
***Steps***	7060 ± 451	6317 ± 480	7334 ± 502	5840 ± 507	62,222 ± 531	3.2	**.017**	0.6	.673	17.6	**<.001**
***No. of sedentary bouts <10 minutes***	28.8 ± 1.6	30.1 ± 1.7	31.2 ± 1.8	29.8 ± 1.8	28.4 ± 1.9	0.7	.627	0.2	.914	.02	.894
***Time in sedentary bouts <10 minutes***	84.8 ± 4.6	88.7 ± 5.0	89.9 ± 5.2	87.9 ± 5.1	84.3 ± 5.2	0.5	.764	0.7	.568	0.1	.814
***No. of sedentary bouts <20 minutes***	35.0 ± 1.8	36.3 ± 1.9	37.6 ± 2.0	36.3 ± 2.0	34.7 ± 2.1	0.6	.654	0.2	.959	0.1	.793
***Time in sedentary bouts <20 minutes***	173.0 ± 7.7	176.7 ± 8.2	180.9 ± 8.6	180.5 ± 8.5	175.4 ± 8.5	0.3	.854	0.3	.886	0.2	.624
***No. of sedentary bouts 11–30 minutes***	9.1 ± 0.4	9.3 ± 0.4	9.4 ± 0.4	9.6 ± 0.4	9.7 ± 0.4	0.7	.612	0.3	.889	0.1	.737
***Time in sedentary bouts 11–30 minutes***	160.9 ± 7.0	163.7 ± 7.5	166.2 ± 7.9	169.2 ± 7.8	173.6 ± 7.8	0.8	.531	0.3	.860	0.1	.822
***No. of sedentary bouts 31–60 minutes***	3.9 ± 0.2	3.6 ± 0.2	4.0 ± 0.2	4.0 ± 0.2	4.0 ± 0.2	1.1	.378	0.8	.518	0.1	.797
***Time in sedentary bouts 31–60 minutes***	163.5 ± 6.4	148.7 ± 7.1	167.5 ± 7.7	165.3 ± 7.5	165.2 ± 7.5	1.3	.265	0.9	.445	.03	.857
***No. of sedentary bouts > 60 minutes***	2.5 ± 0.1	2.8 ± 0.2	2.4 ± 0.2	2.7 ± 0.2	2.5 ± 0.2	1.9	.108	1.3	.284	11.2	**.001**
***Time in sedentary bouts > 60 minutes***	338.6 ± 51.4	362.8 ± 50.0	331.2 ± 53.2	313.3 ± 27.4	347.0 ± 38.2	0.3	.905	0.4	.788	23.0	**<.001**
***No. of sedentary bouts >90 minutes***	1.2 ± 0.1	1.4 ± 0.1	1.2 ± 0.1	1.3 ± 0.1	1.2 ± 0.1	1.1	.368	0.5	.749	12.4	**<.001**
***Time in sedentary bouts > 90 minutes***	244.4 ± 37.1	265.4 ± 50.0	248.8 ± 53.5	204.8 ± 26.3	248.7 ± 38.8	0.5	.754	0.5	.706	20.0	**<.001**
***Total no. of sedentary bouts***	44.3 ± 1.7	45.8 ± 1.8	47.0 ± 1.9	46.1 ± 1.9	44.5 ± 2.0	0.6	.635	0.1	.969	0.0	1.00
***Total sedentary waking time***	12.6 ± 0.6	12.9 ± 0.7	12.5 ± 0.7	12.3 ± 0.7	12.7 ± 0.7	0.1	.967	0.5	.719	12.5	**<.001**

Data are presented as estimated marginal mean ± SE. Abbreviations: LIPA, light-intensity physical activity; MVPA, moderate- to vigorous-intensity physical activity.

Baseline 6MTT results were significantly associated to standing hours (β = 0.006, *P* < 0.001), stepping hours (β = 0.002, *P* = 0.002), LIPA hours (β = 0.001, *P* = 0.009), MVPA hours (β = 0.001, *P* < 0.001), step count (β = 13.3, *P* = <0.001), no. of sedentary bouts >60 minutes (β = −.003, *P* = 0.001), time in sedentary bouts >60 minutes (β = −.778, *P* < 0.001), no. of sedentary bouts >90 minutes (β = −.002, *P* < 0.001), time in sedentary bouts >90 minutes (β = −.716, *P* < 0.001) and total waking sedentary time (β = −.0101, *P* < 0.001).

## DISCUSSION

The PERIOP-OG trial is the first RCT to evaluate a community-based exercise prehabilitation program on activity behavior in esophagogastric cancer patients with curative intent. This study demonstrates that all participants going through this treatment pathway have lower than recommended levels of PA and higher levels of SB. There were no significant differences between the groups for either outcome measures.

The PERIOP-OG trial demonstrates that even before treatment commences, people with this cancer type have lower than recommended PA levels. It would have been interesting to explore at baseline participants pre-cancer diagnosis PA levels to understand these data further (i.e. whether PA levels were reduced due to disease-related effects such as malnutrition or their typical daily PA levels). It is well documented that NCT has a negative impact on physical fitness and quality of life in this patient cohort.^16–18^ However, to our knowledge, no study has investigated PA levels and behaviors via device-based measures in this patient cohort undergoing this complex cancer treatment pathway. Prehabilitation commencing before NCT and continuing during NCT up to the point of surgery can preserve and improve fitness.^19^^,^^20^ To date, prehabilitation has been mainly delivered in hospital settings. A program offered in the community allows for patient to adhere to exercise during NCT, a time when exercise needs to be convenient for the patient for adherence. Targeting patients with an exercise intervention (at home or in a local center) at the time of cancer diagnosis in the PERIOP-OG trial improved physical fitness as patients approached surgery. However, findings reported in this paper suggest that the intervention did not have a positive influence on daily activity behavior outside of the exercise program. It is possible that a compensatory effect existed within the EX group, where after an exercise sessions was completed, these participants may have reduced PA throughout the remainder of the day. Strategies to encourage PA and reduced SB outside of exercise participation may be needed when administering exercise interventions within this cohort.

Our findings are novel and highlight the need for daily PA to be discussed by the medical teams at every point in the cancer care treatment pathway to encourage and empower patients to move more. Our findings highlight that those who had higher baseline fitness had enhanced levels of activity behavior throughout compared to those with lower fitness levels. These data reinforce the need to offer personalized interventions aimed at optimizing PA levels from the start of the cancer diagnosis.^21^

SB was high in both the UC and EX groups. Levels of SB exceeded 12 hours per day throughout the treatment journey for participants in this study. This is consistent with findings in other studies.^22^ High SB is a common observation post-cancer diagnosis and is linked to both cancer recurrence and quality of life, highlighting the importance of reducing SB in this population.^23^ The PERIOP-OG trial showed no significant reduction in SB in EX. This may be attributed to the fact that multiple strategies are required to change behavior rather than solely targeting reducing total SB.^24^ Belcher *et al.* found that studies targeting SB reduction or interruption were more successful than studies with multiple interventions components targeting more than just SB.^22^

Reducing SB may help with the alleviation of cancer symptoms. For the participants involved in this study, approximately half of their daily SB (6 hours/day) was spent in bouts of sitting that lasted >60 minutes. Research has demonstrated that prolonged or less fragmented SB can have detrimental health implications.^25–27^ Lower quality of life has been associated with prolonged SB in cancer cohorts.^28^^,^^29^ Breaking up prolonged SB can have a beneficial effect on indices of health, independent of total sedentary time. Research has reported favorable associations between the number of breaks in sedentary time and measures of adiposity, blood lipids, glucose control, and inflammation.^30–33^ The cancer treatment journey is likely to have a considerable impact on SB as a result of the intense treatment related side effects such as pain and fatigue experienced by the majority of patients.^34^ The evidence suggests that SB may need to be targeted within this cohort, independently of PA. In addition, at certain phases of the cancer treatment journey, reducing SB may be a more attainable goal over increasing exercise participation for this patient cohort completing this complex cancer treatment pathway.

This trial demonstrates that the 6MTT is associated with activity levels as patients who are fitter had higher levels of activity. The 6MTT may be a valid tool in predicting what level patients are at. In breast cancer patients, it was found that the 6MTT can be used to prescribe exercise intensity for this population.^35^ The 6MWT is a valid representative of aerobic fitness.^36^^,^^37^ Research in patients with colorectal cancer reported aerobic fitness to be an independent predictor of risks of complications during surgery.^38^ Maintaining or improving aerobic fitness throughout the cancer treatment journey is a paramount factor in optimizing patient outcomes.^39^

### Strengths and limitations

Patient and public involvement were involved in trial design, development, and oversight, ensuring quality and relevance of the research. Furthermore, each exercise program was tailored to each individual participant: they were provided with activity trackers, were contacted weekly, and constant feedback through exercise logbooks and motivational reviews. Strengths also include the use of validated measure of PA and SB.

There are limitations with this study. The COVID-19 pandemic impacted significantly on this trial. Some monitors got lost in the post during this period, and therefore, no data were obtained for some participants. Recruitment was extended from July 2020 to December 2020 as the trial was paused from March to April 2020 in response to the pandemic, while one center was unable to continue recruitment beyond this point. In addition, due to the nature of this being a nested study as part of a larger randomized, pragmatic, controlled, multi-center, trial (PERIOP-OG Trial) (powered for primary outcome 6MTT), it was not powered to detect statistically significant differences in PA variables. Future work should focus on adequately powered study in this patient cohort.

## CONCLUSION

We demonstrated that commencing a community-based exercise program at cancer diagnosis/pre-NCT and continuing it until the point of surgery showed no significant differences in PA measures compared to UC. The esophagogastric cancer cohort are at risk of low levels of PA with accompanying high levels of SB. Establishing effective interventions to improve activity behavior across this complex cancer treatment journey for this cohort are warranted in future research.
